# Authorization of Animal Experiments Is Based on Confidence Rather than Evidence of Scientific Rigor

**DOI:** 10.1371/journal.pbio.2000598

**Published:** 2016-12-02

**Authors:** Lucile Vogt, Thomas S. Reichlin, Christina Nathues, Hanno Würbel

**Affiliations:** 1 Division of Animal Welfare, Veterinary Public Health Institute, Vetsuisse Faculty, University of Bern, Bern, Switzerland; 2 Division of VPH-Epidemiology, Veterinary Public Health Institute, Vetsuisse Faculty, University of Bern, Liebefeld, Switzerland; Charité—Universitätsmedizin Berlin, Germany

## Abstract

Accumulating evidence indicates high risk of bias in preclinical animal research, questioning the scientific validity and reproducibility of published research findings. Systematic reviews found low rates of reporting of measures against risks of bias in the published literature (e.g., randomization, blinding, sample size calculation) and a correlation between low reporting rates and inflated treatment effects. That most animal research undergoes peer review or ethical review would offer the possibility to detect risks of bias at an earlier stage, before the research has been conducted. For example, in Switzerland, animal experiments are licensed based on a detailed description of the study protocol and a harm–benefit analysis. We therefore screened applications for animal experiments submitted to Swiss authorities (*n* = 1,277) for the rates at which the use of seven basic measures against bias (allocation concealment, blinding, randomization, sample size calculation, inclusion/exclusion criteria, primary outcome variable, and statistical analysis plan) were described and compared them with the reporting rates of the same measures in a representative sub-sample of publications (*n* = 50) resulting from studies described in these applications. Measures against bias were described at very low rates, ranging on average from 2.4% for statistical analysis plan to 19% for primary outcome variable in applications for animal experiments, and from 0.0% for sample size calculation to 34% for statistical analysis plan in publications from these experiments. Calculating an internal validity score (IVS) based on the proportion of the seven measures against bias, we found a weak positive correlation between the IVS of applications and that of publications (Spearman’s *rho* = 0.34, *p* = 0.014), indicating that the rates of description of these measures in applications partly predict their rates of reporting in publications. These results indicate that the authorities licensing animal experiments are lacking important information about experimental conduct that determines the scientific validity of the findings, which may be critical for the weight attributed to the benefit of the research in the harm–benefit analysis. Similar to manuscripts getting accepted for publication despite poor reporting of measures against bias, applications for animal experiments may often be approved based on implicit confidence rather than explicit evidence of scientific rigor. Our findings shed serious doubt on the current authorization procedure for animal experiments, as well as the peer-review process for scientific publications, which in the long run may undermine the credibility of research. Developing existing authorization procedures that are already in place in many countries towards a preregistration system for animal research is one promising way to reform the system. This would not only benefit the scientific validity of findings from animal experiments but also help to avoid unnecessary harm to animals for inconclusive research.

## Author Summary

Scientific validity of research findings depends on scientific rigor, including measures to avoid bias, such as random allocation of animals to treatment groups (randomization) and assessing outcome measures without knowing to which treatment groups the animals belong (blinding). However, measures against bias are rarely reported in publications, and systematic reviews found that poor reporting was associated with larger treatment effects, suggesting bias. Here we studied whether risk of bias could be predicted from study protocols submitted for ethical review. We assessed mention of seven basic measures against bias in study protocols submitted for approval in Switzerland and in publications resulting from these studies. Measures against bias were mentioned at very low rates both in study protocols (2%–19%) and in publications (0%–34%). However, we found a weak positive correlation, indicating that the rates at which measures against bias were mentioned in study protocols predicted the rates at which they were reported in publications. Our results indicate that animal experiments are often licensed based on confidence rather than evidence of scientific rigor, which may compromise scientific validity and induce unnecessary harm to animals caused by inconclusive research.

## Introduction

Reproducibility is a fundamental principle of the scientific method and distinguishes scientific evidence from mere anecdote. The advancement of basic as well as applied research depends on the reproducibility of the findings, and can be seriously hampered if reproducibility is poor. However, accumulating evidence indicates that reproducibility is poor in many disciplines across the life sciences [1]. For example, in a study on microarray gene expression, only 8 out of 18 studies could be reproduced [2]; Prinz and colleagues [3] found large inconsistencies (65%) between published and in-house data in the fields of oncology, women’s health, and cardiovascular diseases; oncologists from Amgen could confirm only 6 out of 53 published findings [4]; and, of more than 100 compounds that showed promising effects on amyotrophic lateral sclerosis (ALS) in preclinical trials, none displayed the same effect when retested by the ALS Therapy Development Institute in Cambridge [5]. Besides a waste of time and resources for inconclusive research [6–8], however, poor reproducibility also entails serious ethical problems. In clinical research, irreproducibility of preclinical research may expose patients to unnecessary risks [9,10], while in basic and preclinical animal research, it may cause unjustified harm to experimental animals [11].

Reproducibility critically depends on experimental design and conduct, which together account for the internal and external validity of experimental results [12]. External validity refers to how applicable results are to other environmental conditions, experimenters, study populations, and even to other strains or species of animals (including humans) [12]. Thus, it also determines reproducibility of the results across replicate studies (i.e., across different labs, different experimenters, different study populations, etc.) [11,13,14]. Internal validity refers to the extent to which a causal relation between experimental treatment and outcome is warranted, and critically depends on scientific rigor, i.e., the extent to which experimental design and conduct minimize systematic bias [12,15]. It has been suggested that poor internal validity due to a lack of scientific rigor may also be a major cause of poor reproducibility in animal research [16–18].

There are various sources of bias (e.g., selection bias, performance bias, detection bias), and specific measures exist to mitigate them (e.g., randomization, blinding, sample-size calculation; [12,15,19,20]). To assess the internal validity of studies, e.g., in the peer review process, and to facilitate replication of studies, publications must contain sufficiently detailed information about experimental design and conduct, including measures taken against risks of bias [20,21]. However, systematic reviews generally found a low prevalence of reporting of measures against risks of bias (further referred to as reporting) in animal research publications. Thus, reporting ranged from 8% to 55.6% for allocation concealment, from 3% to 61% for blinded outcome assessment, from 7% to 55% for randomization, and from 0% to 3% for sample size calculation [19,22–29].

Low rates of reporting have been interpreted as evidence for a lack of scientific rigor (e.g., [20]). Indeed, several systematic reviews found correlations between poor reporting and overstated treatment effects [19,29–31]. Reporting guidelines have thus become a major weapon in the fight against risks of bias in animal research [32]. However, although the ARRIVE guidelines (Animal Research: Reporting of In Vivo Experiments) by the United Kingdom-based organization NC3Rs (National Centre for the Replacement, Refinement & Reduction of Animals in Research) have been endorsed by over 1,000 journals, this did not lead to a substantial improvement of reporting in animal studies [33]. Nevertheless, awareness seems to rise, as Macleod and colleagues [28] recently found that reporting increased over the past decades, although there is still considerable scope for improvement.

Research on the internal validity of animal experiments has focused mainly on reporting in scientific publications. However, most published research has undergone peer review when submitted for funding, and in some countries (e.g., Switzerland, Germany), individual animal experiments are licensed by national or regional authorities. For example, in Switzerland, the licensing of animal experiments is based on an explicit harm–benefit analysis, whereby any harm imposed on the animals is gauged against the expected benefit (gain of knowledge) of the experiment. Because the gain of knowledge critically depends on the scientific validity of the findings, risks of bias may affect the weight attributed to the expected benefit of a study in the harm–benefit analysis. An accurate harm–benefit analysis thus depends on information regarding risks of bias and measures used to mitigate them.

In the present study, we therefore screened applications for animal experiments submitted to the cantonal authorities in Switzerland (*n* = 1,277) for evidence of the use of measures to avoid risks of bias, and compared the rates at which these measures were described in applications (for reasons of simplicity hereafter also referred to as reporting) with the rates of reporting of the same measures in a representative sub-sample of publications (*n* = 50) resulting from experiments described in these applications. This allowed us, for the first time, to compare evidence of scientific rigor available to the authorities when licensing animal experiments with the evidence reported in scientific publications, and to assess whether poor reporting in the scientific literature is predicted by poor reporting in applications for experiments.

## Results

Our database included a final sample of 1,277 applications for animal experiments approved by the cantonal authorities of Switzerland in the years 2008, 2010, and 2012, respectively. Evidence of scientific rigor was assessed based on seven common measures against risks of bias: allocation concealment, randomization, blinded outcome assessment, sample size calculation, inclusion and exclusion criteria, primary outcome, and a statistical analysis plan (S2 and S3 Texts). Besides analyzing each item separately, we also calculated an internal validity score (IVS; see Eq 1), which served as the primary outcome variable for the statistical analysis of effects of various study descriptors on rates of reporting. In addition, we calculated an accuracy score (AS; see Eq 2) based on six items of information explicitly asked for on the application form as a measure of how accurately the applicants had filled out the application form to control for effects of accuracy on the IVS.

### Reporting Rates

Reporting rates were generally very low (Table 1); on average, less than one out of the seven items were reported in applications for animal experiments, with reporting rates varying among the seven items, ranging from 2.4% for the statistical analysis plan to 18.5% for the primary outcome variable (Table 1). However, reporting rates greatly differed between individual applications, with the IVS ranging from 0 (i.e., 0/7 items reported) to 0.857 (i.e., 6/7 items reported), whereby 711 out of the 1,277 applications (55.68%) scored 0 (S1 Fig).

**Table 1:** Reporting rates (in %) of measures against risks of bias in applications for animal experiments in Switzerland depending on year of authorization, type of institution, use of genetically modified animals, authorizing canton, language of the application, species category, and degree of severity of the planned procedures.

Study descriptors		Allocation concealment	Blinded outcome assessment	Random allocation to groups	Formal sample size calculation	Inclusion/ exclusion criteria	Primary outcome	Detailed statistical analysis
	*Overall*	17.62	3.22	12.62	7.91	11.43	18.48	2.35
**Authorization year**	*2008*	17.52	2.40	13.74	5.59	7.45	19.68	2.39
	*2010*	15.22	3.33	12.08	7.09	8.51	16.55	1.42
	*2012*	19.85	3.77	12.21	10.46	17.15	19.25	3.14
**Institution Type**	*Academia*	18.79	3.51	10.96	7.30	9.36	16.98	1.75
	*Industry*	20.99	2.30	21.18	4.60	16.09	24.14	3.45
	*Government*	7.14	1.79	31.11	16.07	21.43	26.79	3.57
	*Other*	11.89	2.47	12.00	10.49	17.90	21.60	4.94
**Genetically modified species**	*Yes*	18.18	2.67	5.95	8.19	6.29	17.33	1.52
	*No*	17.21	3.60	17.40	7.71	15.03	19.28	2.93
**Cantons**	*Small*	13.33	3.60	15.46	12.61	18.92	23.42	5.41
	*1*	21.02	6.52	15.95	5.98	11.96	19.57	5.98
	*2*	21.02	1.05	4.49	9.38	4.69	16.67	0.00
	*3*	10.67	1.40	8.05	3.85	6.64	10.84	0.00
	*4*	21.13	5.07	14.52	9.23	13.99	19.94	1.19
	*5*	21.05	0.96	16.16	2.88	9.62	25.00	5.77
	*6*	11.54	1.56	28.07	20.31	28.13	28.13	4.69
**Languages**	*EN*	24.84	4.07	13.56	6.65	9.61	19.22	2.40
	*DE*	16.61	4.97	17.09	11.57	18.73	25.90	4.68
	*FR*	8.31	0.27	7.06	6.17	6.97	10.19	0.00
**Species categories**	*CDRP*	14.52	15.00	46.15	7.50	41.25	28.45	3.75
	*Farm animal*	14.40	5.11	22.22	12.50	27.27	19.32	5.11
	*Other mammals*	0.00	0.00	40.00	13.0	13.00	38.00	0.0
	*Lab rodent*	19.38	2.18	8.80	7.83	6.53	17.41	1.63
	*Non-mammals*	6.67	0.00	7.89	0.00	4.26	17.02	3.19
**Degree of severity**	*0*	6.67	7.56	18.95	6.72	21.85	19.33	3.36
	*1*	11.39	2.60	14.58	4.40	12.44	18.39	2.85
	*2*	21.70	2.50	11.06	10.63	10.16	17.97	2.03
	*3*	22.40	4.55	10.16	6.06	5.30	20.45	1.52

EN: English; DE: German; FR: French; CDRP: cats, dogs, rabbits, and primates

### Influence of Study Descriptors

We hypothesized that reporting rates and, thus, the IVS might depend on various characteristics of the studies, including the year of authorization (Year), the types of animals used (Species), the severity of the experimental procedures (Severity), the institution conducting the study (Institution), the canton authorizing the study (Canton), and the language in which the application was written (Language), as well as the AS of the application. Generalized linear models in a Bayesian information criterion selection process were used to identify which of the study descriptors best described our data, indicating that they were most likely to have influenced the IVS. The best fitting model included Year, Canton, Language, Institution, and the interaction between Species and AS (see Eq 4). According to the model output (S1 Data), however, none of the individual descriptors had a significant effect on the IVS except Language, as applications written in German had a significantly higher IVS compared to applications written in English (odds ratio [OR] = 0.79, 95% confidence interval [CI] = 0.64–0.98) and applications written in French (OR = 0.46, CI = 0.32–0.65), and the interaction between farm animals and AS (OR = 168.24, CI = 1.17–2,5571.31). Thus, below we report trends that were observed regarding effects of the descriptors that were included in the final model on the IVS.

**Fig 1:**
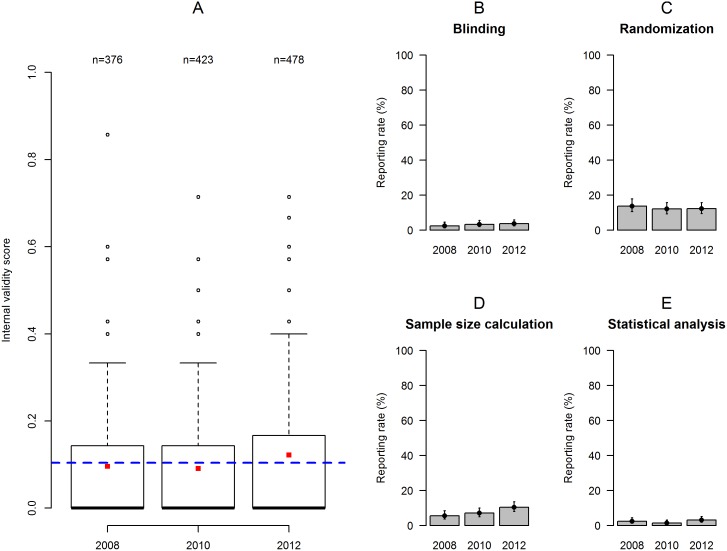
Internal validity score of applications depending on the year of authorization. (A) Boxplot of the IVS for the three years of authorization. The dashed blue line represents the overall mean IVS for the entire sample. The red squares represent the mean IVS for each year. (B–E) Barplots (with binomial confidence intervals) representing reporting rates per year of authorization for blinding (B), randomization (C), sample size calculation (D), and statistical analysis (E). Individual data are shown in https://figshare.com/s/bc48ed5dff9e6ebd2000 (Figure 1).

**Fig 2:**
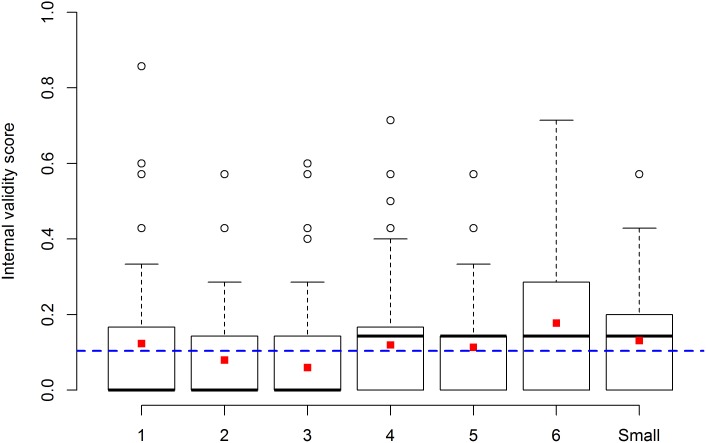
Internal validity score of applications depending on the authorizing canton. Boxplot of the IVS for the six largest cantons (1–6) and the group of small cantons. The dashed blue line represents the overall mean IVS for the entire sample. The red squares represent the mean IVS for each canton or group of cantons. Individual data are shown in https://figshare.com/s/bc48ed5dff9e6ebd2000 (Figure 2).

**Fig 3:**
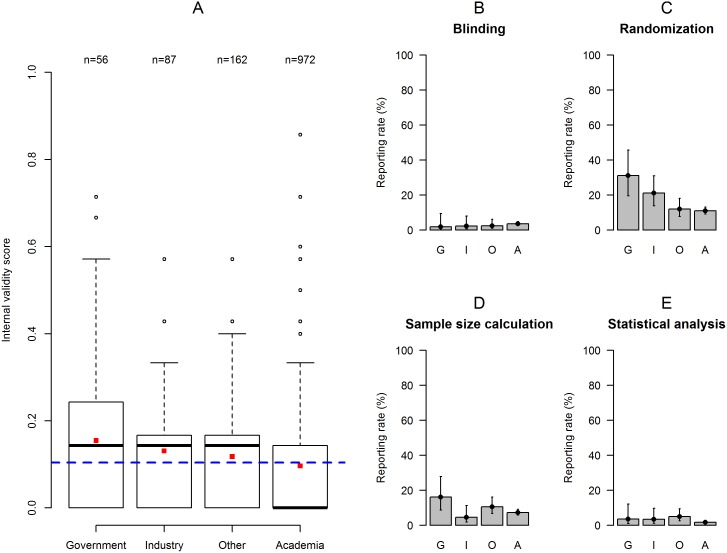
Internal validity score of applications depending on institutions. (A) Boxplot of the IVS for the four categories of institutions. The dashed blue line represents the overall mean IVS for the entire sample. The red squares represent the mean IVS for each category of institutions. (B–E) Barplots (with binomial confidence intervals) representing the reporting rates for each category of institutions (A: academia; G: governmental institutions; I: industry; O: other) for blinding (B), randomization (C), sample size calculation (D), and statistical analysis (E). Individual data are shown in https://figshare.com/s/bc48ed5dff9e6ebd2000 (Figure 3).

**Fig 4:**
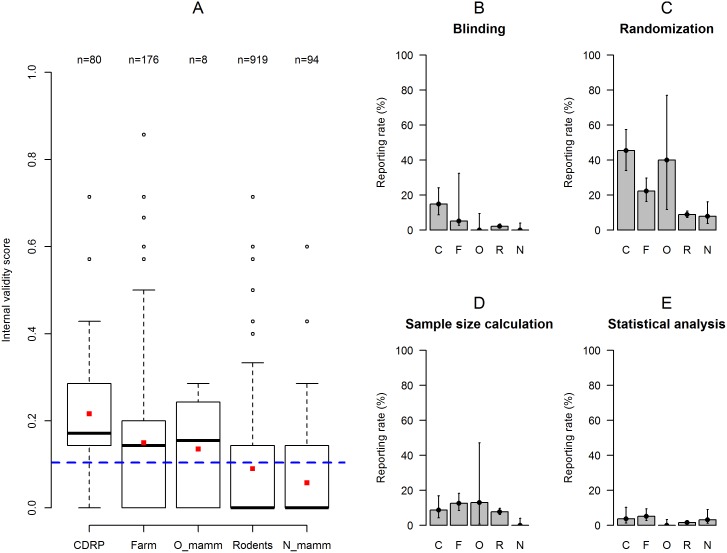
Internal validity score of applications depending on the species of animals. (A) Boxplot of the IVS for the five categories of animal species (CDRP: cats, dogs, rabbits and, primates; Farm: farm animals; O_mamm: other mammals; Rodents: laboratory rodents; N_mamm: non-mammals). The dashed blue line represents the overall mean IVS for the entire sample. The red squares represent the mean IVS for each category of animal species. (B–E) Barplots (with binomial confidence intervals) representing the reporting rates for each category of species (C: cats, dogs, rabbits and, primates; F: farm animals; O: other mammals; R: laboratory rodents; N: non-mammals) for blinding (B), randomization (C), sample size calculation (D), and statistical analysis (E). Individual data are shown in https://figshare.com/s/bc48ed5dff9e6ebd2000 (Figure 4).

The IVS was similar across all three years of authorization: 2012 (median = 0.0, range: 0–0.71), 2010 (0.0, 0–0.71), and 2008 (0.0, 0–0.85). At the level of individual items, trends of improvement across years were observed in the reporting rates of blinding, sample size calculation, and statistical analysis plan (Fig 1B–1E). While there was some variation in IVS across cantons, canton did not seem to have a strong effect (Fig 2). Among the different research institutions, academic institutions (i.e., universities, federal institutes of technology, or university hospitals) accounted by far for the largest part of applications, with 972 (76%) applications compared to 87 (7%) from industry, 56 (4%) from governmental institutions, and 162 (13%) from other private institutions. Overall, academic institutions (0.0, 0–0.86) tended to score lower on IVS than institutions from industry (0.14, 0–0.57), governmental institutions (0.14, 0–0.71), and other private institutions (0.14, 0–0.57; Fig 3A). At the level of individual items, similar trends were observed in the reporting rates of randomization and sample size calculation (Fig 3B–3E). There was also variation in IVS depending on the species of animals used (Fig 4A). Thus, applications for experiments on “higher” mammals (i.e., cats, dogs, rabbits, and primates [CDRP]) tended to score higher (0.17, 0–0.71) compared to experiments on farm animals (0.14, 0–0.86), other mammals (0.15, 0–0.29), laboratory rodents (0.0, 0–0.71), and non-mammals (0.0, 0–0.6), respectively. A similar trend was observed in the reporting rates of blinding, randomization, sample size calculation, and statistical analysis (Fig 4B–4E). Thus, applications for experiments on CDRP as well as farm animals scored higher compared to those involving laboratory rodents and non-mammals, while data from applications for experiments involving other mammals varied widely due to the small sample size (*n* = 8).

**Fig 5:**
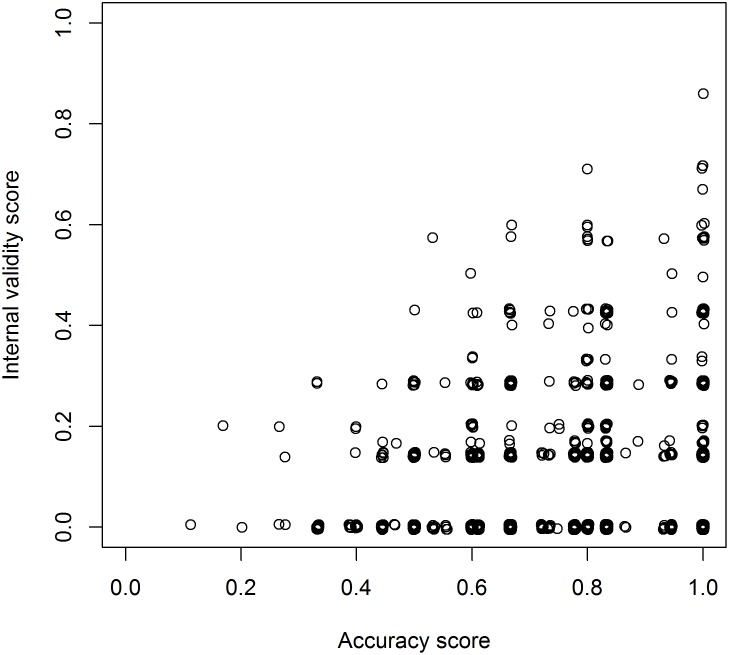
Relationship between AS and IVS. Scatter plots of IVS in relation to AS. Individual data are shown in https://figshare.com/s/bc48ed5dff9e6ebd2000 (Figure 5).

In contrast to the IVS, the AS was generally high, with a median score of 0.8, ranging from 0.11 to 1.00. Despite the low IVS and more than half of the applications scoring 0, there was a weak but positive correlation between AS and IVS (Spearman’s *rho* = 0.17, *p* < 0.001; Fig 5).

### Reliability

In order to ensure reliability of the data between the two investigators (TSR, LV) as well as across time, inter-rater and intra-rater reliability tests were conducted at regular intervals. Inter-rater reliability scores (see Eq 3) of the IVS ranged from 91.4% to 97.1%, while the respective intra-rater reliability scores ranged from 87.1% to 95.7% for TSR and from 94.3% to 97.1% for LV. Similarly, inter-rater reliability scores of the AS ranged from 91.3% to 96.3%, while the respective intra-rater reliability scores ranged from 87.5% to 97.5% for TSR and from 92.5% to 98.8% for LV (see S2 Data).

### Comparison between Applications and Publications

In order to relate the reporting rates obtained from applications for animal experiments to reporting rates found in the scientific literature, we selected 50 publications originating from 50 independent applications in our sample, screened them for the same seven internal validity criteria, and calculated the IVS for each publication using the same method.

**Fig 6:**
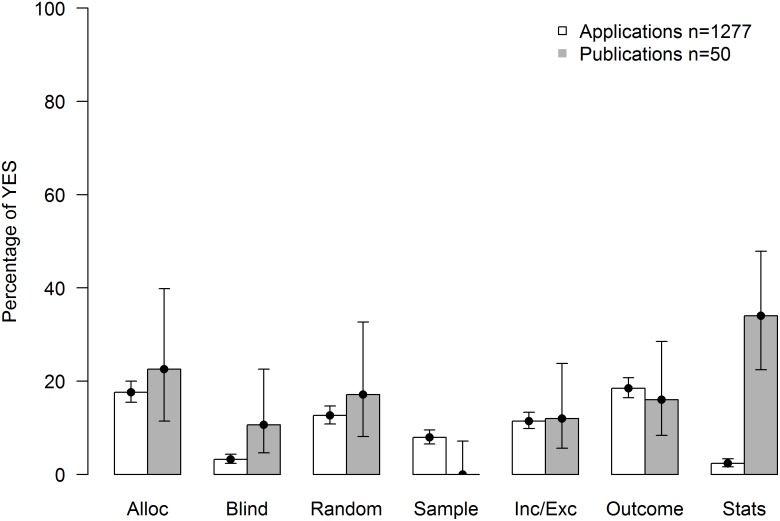
Reporting rates of the seven internal validity criteria in applications and publications. Barplot (with binomial confidence intervals) representing reporting rates of the seven internal validity criteria (Alloc: allocation concealment; Blind: blinding; Random: randomization; Sample: sample size calculation; Inc/Exc: inclusion and exclusion criteria; Outcome: primary outcome; Stats: statistical analysis). Individual data are shown in https://figshare.com/s/bc48ed5dff9e6ebd2000 (Sample Applications and Sample Publications).

Similar to what we found for applications, reporting rates in the 50 publications were generally low, albeit slightly higher than in the applications (Fig 6), resulting in a median IVS of 0.14. Reporting rates for the seven items ranged from 0% for sample size calculation to 34% for the statistical analysis plan. Again, reporting rates differed greatly between individual publications, with IVS ranging from 0 to 0.6, whereby 23 out of 50 publications (46%) scored 0.

Except for sample size calculation and the primary outcome variable, reporting rates for individual items were higher in publications than in applications (see Fig 6). Whereas IVS of applications and publications were the same in 27 cases (of which 21 scored 0), it was higher in 18 pairs (which was due to a statistical analysis plan in 12 cases) and lower in five cases. This increase was corroborated by a weak positive correlation between the IVS of applications and that of publications (Spearman’s *rho* = 0.34, *p* = 0.014).

### Influence of Study Descriptors

**Fig 7:**
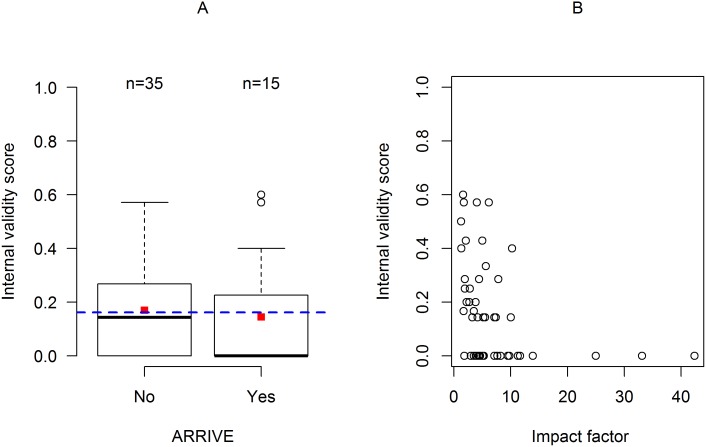
Internal validity score of publications depending on endorsement of the ARRIVE guidelines by the journal and the journal’s impact factor. (A) IVS depending on endorsement of the ARRIVE guidelines. The dashed blue line represents the overall mean IVS for the entire sample of publications. The red squares represent the mean IVS for each group. (B) Scatter plot of the IVS depending on the impact factor of the journal. Individual data are shown in https://figshare.com/s/bc48ed5dff9e6ebd2000 (Sample Publications).

Due to the smaller sample size, not all descriptors assessed for their effects on the IVS of applications could be analyzed here. Instead, we analyzed publication-specific descriptors, namely whether or not the journal in which the study was published had endorsed the ARRIVE guidelines and the impact factor of the journal (IF). There was no significant effect of ARRIVE on IVS (yes: median = 0.14, range: 0 to 0.57; no: median = 0, range: 0 to 0.60; *p* = 0.69; Fig 7A). In contrast, IF had a significant negative effect on IVS (Spearman’s *rho* = -0.49, *p* < 0.001; Fig 7B).

## Discussion

Based on the low reporting rates in publications of animal research and evidence suggesting that poor reporting may reflect a lack of scientific rigor [19,29–31], this study examined whether poor reporting in the scientific literature is predicted by poor reporting in applications for animal experiments, that is before the studies have actually been conducted. The study was restricted to animal experiments authorized in Switzerland for two reasons. First, Switzerland has an authorization system for animal experiments that requires detailed description of study protocols for every planned study. These study protocols form the basis of the harm–benefit analysis upon which the decision for or against authorization of individual studies is based. Second, the study was facilitated by the Swiss Federal Food Safety and Veterinary Office (FSVO) providing access to all applications for animal experiments via their online platform (e-tierversuche) through which scientists communicate with the authorities and submit their applications for animal experiments. Such unlimited access to application forms for animal experiments is unprecedented, and it is laudable that the FSVO supported this meta-research. This kind of support has notoriously proven difficult to obtain for reasons of confidentiality, as highlighted by Chan et al. [34], with respect to clinical trial protocols for meta-research. As described in the Materials and Methods, access to the application forms was possible without violating confidentiality.

### Low Reporting Rates

We evaluated 1,277 applications for animal experiments and 50 publications derived thereof and found very low reporting rates in both applications and publications (Fig 6). Reporting rates in publications were within the range reported in previous studies (e.g., [19,20]). That reporting rates in applications were similar—even slightly lower—indicates that the authorities approving animal experiments are lacking important information about experimental conduct that may be critical for evaluating the expected benefit in a harm–benefit analysis. Risks of bias question the scientific validity of the results, which is a precondition for a study to achieve the expected benefit. Whether the authorities are unaware of risks of bias and measures to avoid them or whether they consider them as unimportant for the benefit of the research is unknown and warrants further study. As a result, however, animal experiments are authorized based on implicit confidence rather than explicit evidence of scientific rigor. Similarly, poor reporting in publications means that manuscripts are often accepted for publication in the absence of evidence of scientific rigor. This “trust me model” of science has been criticized before [1,35,36]. It sheds serious doubts on the current authorization procedure for animal experiments as well as the peer-review process for scientific publications, which in the long run may compromise the credibility of the research.

### Relationship between Reporting in Applications and Publications

We found a weak positive correlation between the IVS of applications and that of the corresponding publications. This suggests that the reporting of bias avoidance measures in applications predicted, at least to some extent, the reporting of such measures in publications. If this reflects a consistent relationship, asking for more detailed information on experimental conduct in applications for animal experiments might help to promote better experimental conduct as well as better reporting in publications. Asking for more detailed information at the planning stage of the research might also reduce the danger of normative responses, whereby scientists simply satisfy the guidelines (e.g., ARRIVE) at a time when it is too late to take corrective actions on experimental conduct.

The increase in the IVS of publications compared to applications was largely due to better reporting of the statistical analysis plan (S2 Fig). This is likely due to the fact that journals (and reviewers) generally insist on a detailed description of the statistical analysis. It indicates that reporting guidelines (such as ARRIVE) could potentially increase scientific quality of animal research, if editors and reviewers helped to enforce them. However, as shown by Baker et al. [33] and confirmed by the present study (Fig 7A), this has not been the case so far; publications in journals having endorsed the ARRIVE guidelines did not score higher than publications in other journals. We also found a weak positive correlation between the accuracy of completing the application forms (AS) and the IVS. Thus, applicants who answered questions in the application form more accurately had a higher IVS. As shown by Minnerup et al. [37], this further confirms that enforcement of guidelines may be important in view of improving reporting standards.

### Effects of Study Characteristics on the IVS of Applications and Publications

In the final statistical model, language was the only descriptor having a significant effect on IVS of applications for animal experiments. Applications written in German had significantly higher IVS than applications written in English or French. Several explanations may account for this result. For example, the proportion of German native speakers may have been higher among authors of German applications; German may have been mostly used by native German speakers, while English may have been used by many non-native English speakers. Similarly, French may have been used by many non-native French speakers because, apparently, authorities in French-speaking cantons of Switzerland strongly encourage submission of applications in French (own observation). However, one might not necessarily expect language skills to affect such standardized terminology (randomization, blinding, etc.), but because these items are not explicitly asked for, applicants writing in their native language might be more likely to provide unsolicited detail. Alternatively, differences in regional policies of authorities between French- and German-speaking cantons, as well as the fact that all French applications were scored by only one experimenter (LV), may have contributed to this effect, but our data do not allow us to examine these explanations further.

Apart from language, all other explanatory variables in the final model had only weak effects on IVS that did not reach statistical significance (S1 Data). For example, there was a weak tendency for the reporting rates of blinding, sample size calculation, and statistical analysis to be higher in 2012 compared to those from previous years (Fig 1). This trend might reflect increasing awareness by both researchers and authorities of the importance of reporting, and it is consistent with recent evidence from a random sample of life sciences publications [28]. However, despite the many systematic reviews revealing flaws in experimental design and conduct since Ioannidis’ seminal opinion paper [38], and the wealth of solutions that have since been proposed [2,5,32,39], little progress has been made. Like Baker et al. in 2014 [33], we did not find convincing evidence that reporting had increased from applications authorized before (2008) to those authorized after (2012) publication of the ARRIVE guidelines. Again, the main reason for this might be a lack of enforcement of these guidelines by authorities as well as journal editors. However, our sample was mostly based on studies designed and authorized before the ARRIVE guidelines became widely known. That the endorsement of the ARRIVE guidelines had no effect on the IVS of publications may thus reflect the delay in such a change taking effect.

Recent evidence indicated that industry-sponsored research is less biased than academic research [40]. We therefore predicted higher rates of reporting of measures against risks of bias in applications from private compared to academic institutions. Although there was a weak tendency for applications from academic institutions to score lower on IVS compared to governmental and private institutions, we cannot exclude random variation as the source of this trend. If true, however, it might reflect the different incentives between institutions, favoring more conservative approaches in non-academic institutions [41].

An interesting tendency was found in relation to the type of animals being used. Thus, applications for experiments on CDRP, farm animals, and other mammals had slightly higher IVS than those for experiments on lab rodents and non-mammals. CDRP and, to a lesser extent, farm animals and other mammals may benefit from the attribution of a higher moral status, e.g., because they are close relatives (primates), social partners (dogs, cats, rabbits), or otherwise elicit more compassion (farm animals, other mammals) than lab rodents (that are also considered as “pest” species) and non-mammals (mostly fish; e.g., [42,43,44]). On the one hand, this might indicate that applications are assessed more carefully when the stakes are perceived as morally high, although it would remain unclear whether this effect is due to the applicants providing more information or to the authorities asking for more. On the other hand, IVS was low throughout, and the difference between species categories was not significant. In addition, there was no such trend with increasing degree of severity of studies. Importantly, however, the Swiss Animal Welfare Act does not provide a legal basis for such “speciesism” among vertebrates, and both authors and authorities should treat all vertebrates equally.

Finally, we found a weak but significant negative relationship between the IVS of publications and the IF of the journal in which it was published. That the journal IF does not necessarily reflect the quality of research has long been known (e.g., [45]), and a systematic review of a random sample of life sciences publications recently found no evidence for a positive relationship between IF and reporting [28]. Across the whole range of journal IF in our sample of publications, IVS of 0 clearly prevails, confirming that poor reporting of measures against risk of bias is common throughout the scientific literature.

### General Conclusions

According to the Animal Protection Index (API) by World Animal Protection, Switzerland (together with the United Kingdom, Austria, and New Zealand) ranked top in an international comparison of animal protection policy among 50 countries (http://api.worldanimalprotection.org/). In particular, authorization of animal experiments is based on a harm–benefit analysis, and authorization is denied if, in relation to the anticipated gain in knowledge, they inflict disproportionate harm on the animals (Article 19(4), [46]). Because the anticipated gain in knowledge critically depends on experimental design and conduct, the lack of information on measures against risks of bias in applications means that, in Switzerland, authorization of animal experiments is based on implicit confidence rather than explicit evidence of scientific rigor.

Several arguments may be held against this interpretation of our results, namely (i) that the measures against risks of bias assessed here are not important determinants of scientific validity, (ii) that they are not explicitly asked for on the application form for animal experiments, (iii) that, as the system currently works, it is not the authorities’ duty to assess the scientific validity of the experiments, and (iv) that the authorities’ confidence in scientific rigor is well justified. First, it is certainly the case that the authorities assess the scientific rationale underlying the proposed studies, thereby assessing several important aspects of scientific validity, although these are not specified explicitly. Also, there may be other, even more important risks of bias (e.g., use of inappropriate control group) that were not included in our evaluation. However, all seven items included here are considered as relevant measures against risks of bias that may compromise scientific validity in important ways; they have therefore been included in reporting guidelines such as the ARRIVE guidelines. Second, while it is also true that the application form does not explicitly ask for allocation concealment, randomization, blinding, and inclusion or exclusion criteria, it does ask explicitly for the primary and secondary outcome variables, sample size calculation, and a detailed statistical analysis plan. Moreover, the first example of how to describe procedures presented in the explanatory notes to the application form by the FSVO starts with “The dogs are divided randomly into 3 groups,” indicating that randomization is also considered a relevant aspect of the description of procedures. Even if only those measures explicitly asked for on the application form were enforced, all applications would score IVS ≥ 0.42 (i.e., 3/7). Third, authorities may argue that it is the peers’ duty to assess and guarantee scientific rigor, while the authorities’ duties (and those of their advisory committees) should be limited to assessing the scope for applying the 3Rs (replacement of animal experiments, reduction of animal use, and refinement of procedures) and whether the expected benefits (as declared by the applicants) outweigh the harms inflicted on the animals. However, it is important to note that not all experiments are based on project proposals that have undergone scientific peer review (e.g., most applications from the private sector), and that peer review does not seem to guarantee good scientific practice [47]. Finally, whether the authorities’ implicit confidence in the scientific validity of the results of licensed experiments is justified is an empirical question. Concerns that such confidence may not be warranted is largely based on studies showing a negative relationship between reporting of measures against risks of bias and inflation of treatment effect size in preclinical studies (e.g., [19,25]). Together with accumulating evidence of poor reproducibility of in vivo research, these findings have shed doubts on the quality of experimental design and conduct. However, there is clearly a need for more research on the actual implementation of measures against risks of bias in experimental animal research. We have recently conducted an online survey amongst all Swiss animal researchers to elucidate actual implementation of the same seven measures against risks of bias assessed here. Our findings suggest that although reporting rates found in the literature tend to underestimate actual implementation of these measures, there is considerable scope for improvement [48].

Lack of scientific rigor in experimental conduct is widely considered to be an important determinant of poor reproducibility of in vivo research [16,17,18]. However, this assumption is based on the indirect evidence outlined above, and has never been tested directly. Randomization, blinding, sample size calculation, and all the other measures against risks of bias assessed here mainly affect the internal validity of experiments. Although the reproducibility of results can be affected by the internal validity of studies, reproducibility depends more on the external validity of studies [11–13]. Reproducibility may thus be enhanced mainly by using design features aimed to increase the external validity of results, such as more heterogeneous study populations, independent replicate cohorts, or multicenter study designs [14,49,50]. Thus, there is also a need for more research on the relative contribution of experimental conduct and experimental design, respectively, to the reproducibility of results.

Last, but not least, besides experimental design and experimental conduct, several other factors introduce bias into the scientific literature, in particular “hypothesizing after results are known” (HARKing, [51]), *p*-hacking [52], selective reporting [53], and publication bias [54]. The most effective way of eliminating all of these biases would be prospective registration of preclinical animal experiments similar to preregistration of clinical trials [55]. Further research is certainly needed on how to facilitate practical implementation of preregistration in the face of several contentious issues such as confidentiality, property rights, and theft of ideas. However, the authorization procedure for animal experiments already in place in Switzerland (and other countries, e.g., Germany), provides an ideal basis for implementing preregistration of animal experiments, which would not only benefit the scientific validity of results from animal experiments but also minimize unnecessary harm to animals for inconclusive research. By this, Switzerland could consolidate its position as a leader in animal protection as well as extend its leadership to scientific rigor.

## Materials and Methods

### Sampling Process

Applications for animal experiments (Form A, S1 Text) were selected from an anonymized database obtained from the FSVO, containing all applications submitted in Switzerland since 1983. Access to applications archived by the FSVO was based on a contract between the FSVO and the authors of this study, which guaranteed confidentiality to the applicants. Applications were selected based on predefined inclusion and exclusion criteria. Thus, only new applications submitted during the years 2008, 2010, and 2012 were included, of which applications related to (i) diagnosis of disease, (ii) education and training, and (iii) the protection of humans, animals, and the environment by toxicological or other safety tests required by law were excluded a priori (S3 Fig). A total of 1590 applications met these criteria and were subjected to formal screening.

### Checklist

In order to assess risks of bias in the experiments described in the applications, a checklist was elaborated (S2 Text) based on checklists used in previous studies assessing the use of measures to reduce risks of biases as reported in the published literature [19,20,56]. We restricted our checklist to items that (i) are essentially applicable to all kinds of experimental studies and (ii) can be assessed objectively without specific expertise of the research topic, and included those seven items that we encountered most often in the literature: (1) allocation concealment, (2) blinded outcome assessment, (3) randomization, (4) formal sample size calculation, (5) inclusion and exclusion criteria, (6) a primary outcome variable, and (7) a statistical analysis plan. These seven items were also used to calculate an IVS based on the number of items that were reported in the application divided by the total number of items applicable to the study (max = 7).

Internal Validity Score=number of items reported /total number of items (1)

Additional items were assessed that were, however, not included in the IVS. These included additional aspects of study conduct (blinded conduct of study, randomized conduct of study, termination criteria, references for the sample size, and general statements on statistical analysis; S2 Text).

In addition, we assessed the accuracy with which the application forms (Form A) were filled out, using items that were explicitly asked for on Form A, and for which the content to be filled in was explicitly specified in the accompanying guidelines to Form A on the FSVO webpage (https://www.blv.admin.ch/dam/blv/en/dokumente/tiere/publikationen-und-forschung/tierversuche/erlaeuterungen-form-a.pdf.download.pdf/erlaeuterungen-form-a.pdf). Furthermore, we chose items that are relevant for the harm–benefit analysis and could be determined with high reliability. The following six items were included: (1) description and justification of the methods used (e.g., by indicating references, previous results, or results from a pilot study); (2) information about the identification of individual animals; (3) the total number of animals used, the number of treatment groups, and the number of animals per treatment group; (4) reference to a score sheet for the assessment of animal welfare; (5) the degrees of severity for all animals involved in the experiments; and (6) the fate of the animals at the end of the experiments. These six items were used to calculate an AS based on the number of items reported divided by the total number of items applicable to the study (max = 6).

Accuracy Score =number of items reported /total number of items (2)

The AS was constructed as a control measure, to control for variation in IVS induced by variation in the accuracy with which the form was filled out. Both IVS and AS were assessed by scoring whether or not the respective items were reported in any of the experiments included in an application form. Thus, a “YES” was recorded if an item was reported in at least one of the described experiments and a “NO” if an item was either not reported at all or if it was unclear. If an item was not applicable to the experiment described in the application form, “NA” was recorded (more details are given in the S3 Text).

### Data Collection

The 1590 applications were randomly allocated to two investigators (LV, TSR) for formal screening (leading to two lists of 795 applications each, one for each investigator). During screening, 94 applications were excluded because they were either incomplete or not available in the archives of the FSVO. A further 36 applications were excluded because they met one or more of the exclusion criteria reported above. This left 1,460 applications that were deemed suitable for screening. Applications written in French (*n* = 423) or Italian (*n* = 5) were screened by the investigator with better knowledge of these languages (LV), regardless of their assignment to the two investigators, while applications written in German (*n* = 430) or English (*n* = 602) were screened according to their assignments to the two investigators. Therefore, a total sample of *n* = 935 was screened by investigator LV while a total sample of *n* = 525 applications was screened by investigator TSR.

To restrict analysis to experimental in vivo studies, a further 183 applications were excluded in the course of the screening process because they referred to in vitro studies (if the animals were killed before the experimental treatment was applied; *n* = 106), monitoring studies (if the animals were observed in the wild; *n* = 28), or other exceptions (e.g., breeding studies, post-mortem studies; *n* = 49), resulting in a final sample size of *n* = 1,277 applications used for analysis (see S3 Fig).

Based on information provided by the applicants on Form A and used for the annual statistics of animal use by the FSVO, we also recorded several descriptors that might influence the reporting of internal validity items; these included (i) year of authorization (2008, 2010, 2012), (ii) language (English, German, French), (iii) canton (the six largest cantons of Basel, Bern, Freiburg, Geneva, Vaud, Zurich, and the group of the remaining small cantons), (iv) type of institution (academic institutions [i.e., universities, federal institutes of technology, hospitals], industry, governmental institutions [national and cantonal], other [e.g., private institutions, foundations]), (v) animal species (laboratory rodents, higher mammals [CDRP], farm animals, other mammals, non-mammals), (vi) genetically modified animals (yes, no), and (vii) the prospective degree of severity of the planned procedures as defined by the FSVO (0, 1, 2, 3).

### Inter-rater Reliability

Prior to the screening of the selected Form A, two pilot studies on separate applications (i.e., applications authorized in 2009) were conducted to ensure the applicability of the checklist and to ensure consistency of scoring within and between investigators. To ensure consistent scoring of applications between the two investigators, both investigators screened the same 10 applications, and discrepancies were checked at the end of the day. Inter-rater reliability (Eq 3) was assessed at regular intervals (on day 1 and then after the 100th, 300th, 500th, and 700th application on the investigators’ list, respectively) by assessing the proportion of agreement between the two investigators. For this, the first five applications on each investigator’s list were screened by both investigators.

Reliability=(no. of items scored−tot. no. of discrepancies)/no. of items scored * 100) (3)

Only applications written in either German or English were used for inter-rater reliability tests. Overall, 50 applications were screened twice in the course of these inter-rater reliability tests. Inter-rater reliability never dropped below 85% (S2 Data).

### Intra-rater Reliability

To ensure that both investigators scored applications consistently over time, samples of 10 applications were re-scored at regular intervals (after 50, 150, 350, and 550 listed applications, respectively). In addition, each investigator conducted a final intra-rater reliability test on 10 randomly chosen applications from the whole list after completing the screening procedure. If systematic discrepancies would have occurred, the applications previously scored would have been re-scored. However, as in the case of inter-rater reliability, intra-rater reliability never dropped below 85% (S2 Data).

### Sample Size Calculation

No a priori sample size calculation was performed, as all applications were included in our sample that fulfilled the inclusion/exclusion criteria. However, once the sample size was determined, we verified that it was suitable for the planned statistical analysis (see model description below).

### Statistics

The screening data from the checklists were transferred to a tabulating program (Microsoft Excel 2010.Ink, Redmond, WA, USA) and imported into the statistical software R [57].

We used descriptive statistics to represent reporting rates for individual criteria of internal validity (allocation concealment, blinded outcome assessment, randomization, sample size calculation, inclusion and exclusion criteria, primary outcome, and statistical analysis). Furthermore, influences of relevant descriptors (year, canton, institution, and animal species) were represented graphically, with median and mean IVS of the group, and overall mean IVS.

For the statistical analysis of the overall internal validity score of applications, we used generalized linear models to evaluate the influence of the a priori stated descriptors on the internal validity score. The analyses were performed in R [57] using the built in function *glm* with a binomial error distribution to account for the data structure (primary outcome as proportions). As a first step, we compared univariate models (model with one descriptor) with an intercept-only model (modelling the intercept of the internal validity score) based on significant (*p* < 0.05) likelihood ratio test of the package lmtest [58] in order to identify descriptors to be included in the further modelling process. The descriptors to be retained were language, canton, species category, institutions, authorization year, and accuracy of the application. In a second step, by means of an information theoretic approach to model selection using the Bayesian Information Criterion (BIC), we identified the model that best fit our data. For an automated model selection procedure, the package MuMln [59] with the function *dredge* was used to compare all models with all possible combinations of the retained descriptors (full model included also the interaction term for species category and accuracy; see Eq 4).

glm(formula=IVS~Language+Canton+Species Category+Accuracy+Institution+Authorization Year+Species Category:Accuracy, family=quasibinomial, weights=maxIVS) (4)

The *dredge* function ranks all descriptor combinations according to their BIC; the model with the lowest BIC was assumed to be the one representing our data best. The final model included the following main effects (descriptors): language (3 levels), cantons (7 levels), species category (5 levels), accuracy (continuous), institution (4 levels), and authorization year (3 levels). In addition to these main effects, the candidate model included the two-way interaction between species category and accuracy (corresponds to full model, cf. Eq 4). The model parameters were retrieved after correction for over dispersion (see S1 Data).

### Publications Sampling Process and Screening

In order to relate the reporting rates of internal validity criteria assessed here by scoring applications for animal experiments with the reporting rates of such criteria in the published literature [19,22–28,60], we also scored a sub-sample of publications originating from studies based on applications in our study sample. These were identified by searching through grant numbers mentioned in the applications and references listed as output in the annual reports to the FSVO. For 155 applications (12.1%) we identified one or more corresponding publications. This number was reduced to 139 after excluding reviews and publications that were clearly unrelated to the study described in the applications (mismatch in animal species, general topic, or methods). This low number can be explained by the fact that studies licensed in 2012 and also many of those licensed in 2010 were not yet published, and that the search for publications had to rely on grant numbers mentioned in both application and publication (often grant numbers were not mentioned on applications) or on publications listed in the final reports required by the authorities upon completion of licensed studies (for most studies licensed in 2012 and also many of those licensed in 2010, final reports were not yet available).

For the comparison of the internal validity scores between applications and publications, we aimed to detect a medium effect size (0.3) with a statistical power of 0.8 at a significance level of *p* < 0.05. Based on this, we chose a sample size of *n* = 50, which allowed us to detect an effect size of 0.276 (G*power for correlations, bivariate normal model) [61]. A stratified random sampling procedure was used to select 50 publications from the 139 available publications, so as to select publications derived from a representative sample of all applications with respect to canton and type of animals used. Because this sample of publications was biased towards older applications, we compared the IVS of the sub-sample of 50 applications from which these 50 publications originated with the IVS of the entire sample of applications and found no significant difference; median IVS of the entire sample of applications *(n* = 1,277) was 0.0 (range 0 to 0.857), compared to 0.0 (range 0 to 0.714) for the sub-sample of applications (*n* = 50) from which the 50 publications were derived.

The publications were screened for reporting of internal validity criteria with a checklist containing the same seven internal validity criteria as were used for applications. The screening of all 50 publications was performed by one single investigator (LV). Publications were randomly allocated to one of the 10 d of screening (five publications per day). Days of screening were separated by two non-screening days. For the publications, descriptors were impact factor of the journal and endorsement of the ARRIVE guidelines by the journal. To determine the descriptors, the impact factor for the year of the publication as well as the ARRIVE status of the journal were assessed. If it was not possible to determine the ARRIVE status of a journal for the date of publication, given that all publications were published in 2012 or later, we used the ARRIVE status of the journal in 2015. Whether or not the ARRIVE status affected the internal validity score of publications was tested with a univariate generalized linear model (binomial error distribution), with IVS as dependent and the descriptor (endorsement of ARRIVE yes or no) as independent variables.

glm(formula=IVS~Endorsement of the ARRIVE, family=quasibinomial, weights=maxIVS) (5)

Whether or not the internal validity score of publications was correlated with the impact factor of the journal was investigated using a spearman rank correlation test.

To ensure that the investigator scored the publications constantly over time, an independent person randomly chose one publication per five publications screened (i.e., one per day of screening) for an intra-rater reliability test. The chosen publication was re-screened on the second following day. The reliability (Eq 3) never dropped below the threshold of 85%.

## Supporting Information

S1 Fig Distribution of internal validity score. Individual data are shown in https://figshare.com/s/bc48ed5dff9e6ebd2000 (Sample Applications).
(TIFF)

S2 Fig Comparison of reporting of internal validity criteria between applications and resulting publications. AC: Allocation concealment, BL: Blinding, RA: Randomization,SS: Sample size calculation, IE: Inlcusion/exclusion criteria, PO: Primary outcome, SA: Statistical analysis. Individual data are shown in https://figshare.com/s/bc48ed5dff9e6ebd2000 (Sample Applications and Sample Publications).
(TIF)

S3 Fig Criteria of inclusion of the application in our study. (TIF)

S1 Text Form A. (PDF)

S2 Text Checklist. (PDF)

S3 Text Annexe to the checklist. (PDF)

S1 Data Outcome of the generalized linear model. Output from the generalized linear model used to identify factors influencing IVS of applications. Data are presented with estimate, odds ratios, and the values for the 2.50% quartile and the 97.5% quartile. For more information about the equation we refer to Materials and Methods.
(XLSX)

S2 Data Outcome of the reliability tests. Outcome of the reliability tests (inter-rater reliability and intra-rater reliability). Percentage agreement and number of discrepancies are available for each item composing the IVS, as well as for the IVS. For more information about the equation we refer to Materials and Methods.
(XLSX)

## Abbreviations

ALS: amyotrophic lateral sclerosis
API: Animal Protection Index
ARRIVE: Animal Research: Reporting of In Vivo Experiments
AS: accuracy score
CDRP: cats, dogs, rabbits, and primates
CI: confidence interval
FSVO: Swiss Federal Food Safety and Veterinary Office
IF: impact factor
IVS: internal validity score
NC3Rs: National Centre for the Replacement, Refinement & Reduction of Animals in Research
OR: odds ratio
