# Supplementary figures and images for: Authorization of Animal Experiments Is Based on Confidence Rather than Evidence of Scientific Rigor
Source: PLoS Biol. 2016 Dec 2;14(12):e2000598. doi: 10.1371/journal.pbio.2000598 (PMC5135031; doi:10.1371/journal.pbio.2000598)

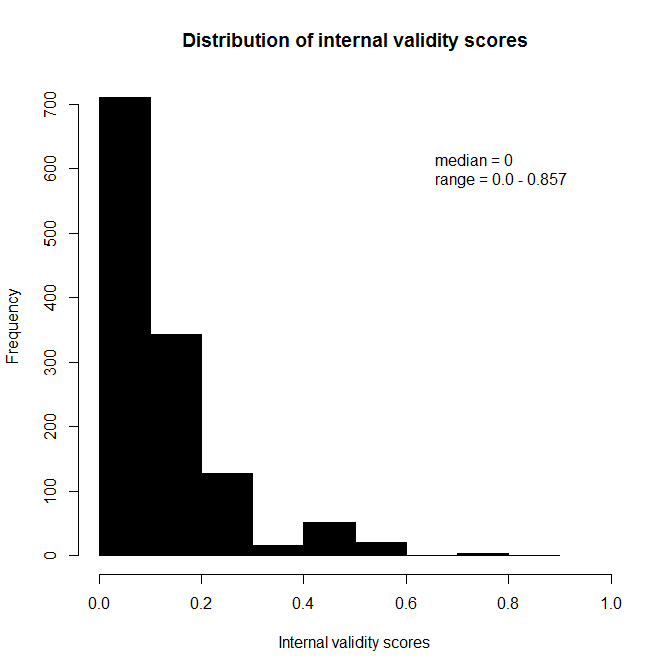

Supplement: S1 Fig — Individual data are shown in https://figshare.com/s/bc48ed5dff9e6ebd2000 (Sample Applications). (TIFF) [file pbio.2000598.s001.tiff]

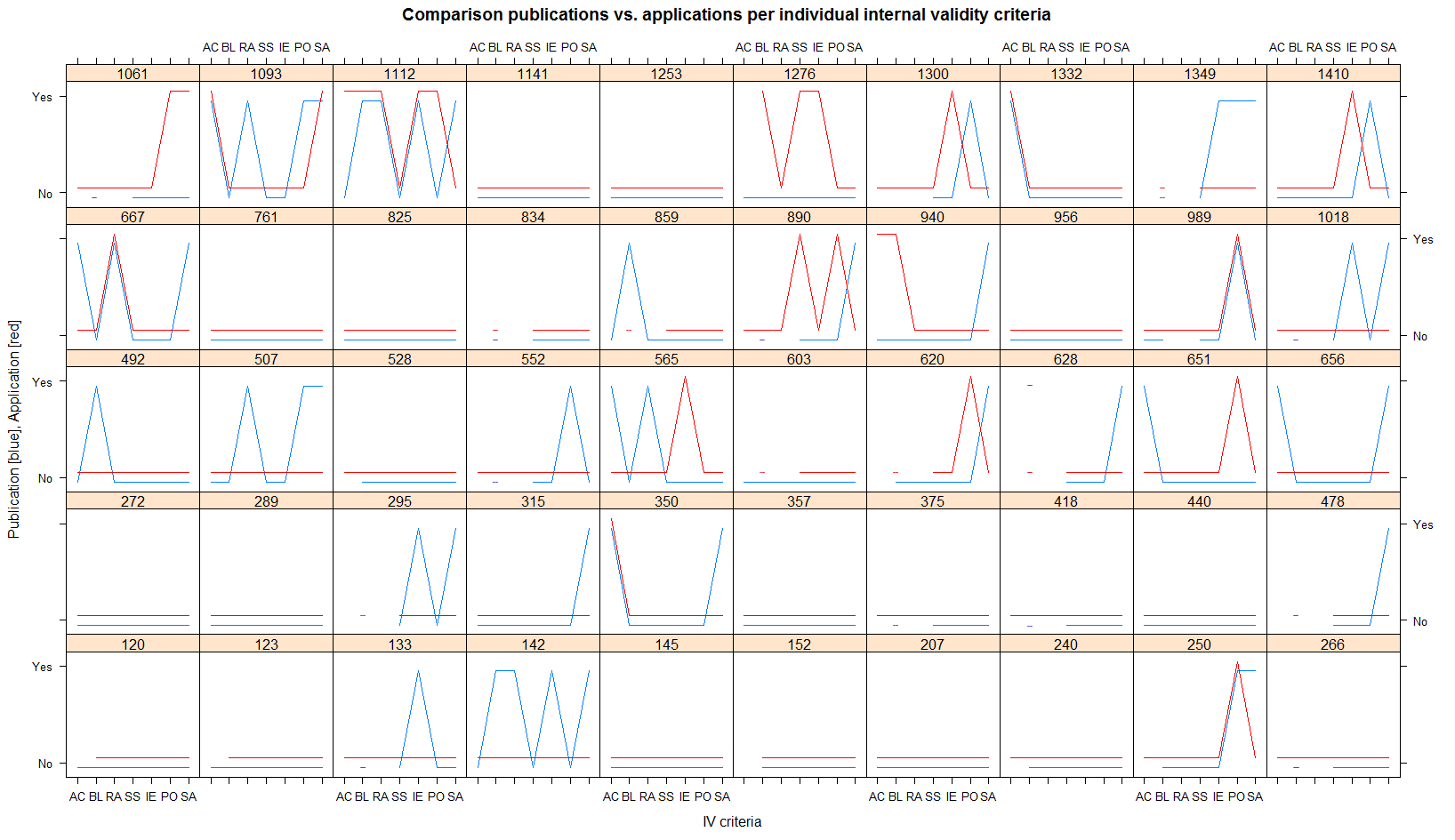

Supplement: S2 Fig — AC: Allocation concealment, BL: Blinding, RA: Randomization,SS: Sample size calculation, IE: Inlcusion/exclusion criteria, PO: Primary outcome, SA: Statistical analysis. Individual data are shown in https://figshare.com/s/bc48ed5dff9e6ebd2000 (Sample Applications and Sample Publications). (TIF) [file pbio.2000598.s002.tif]

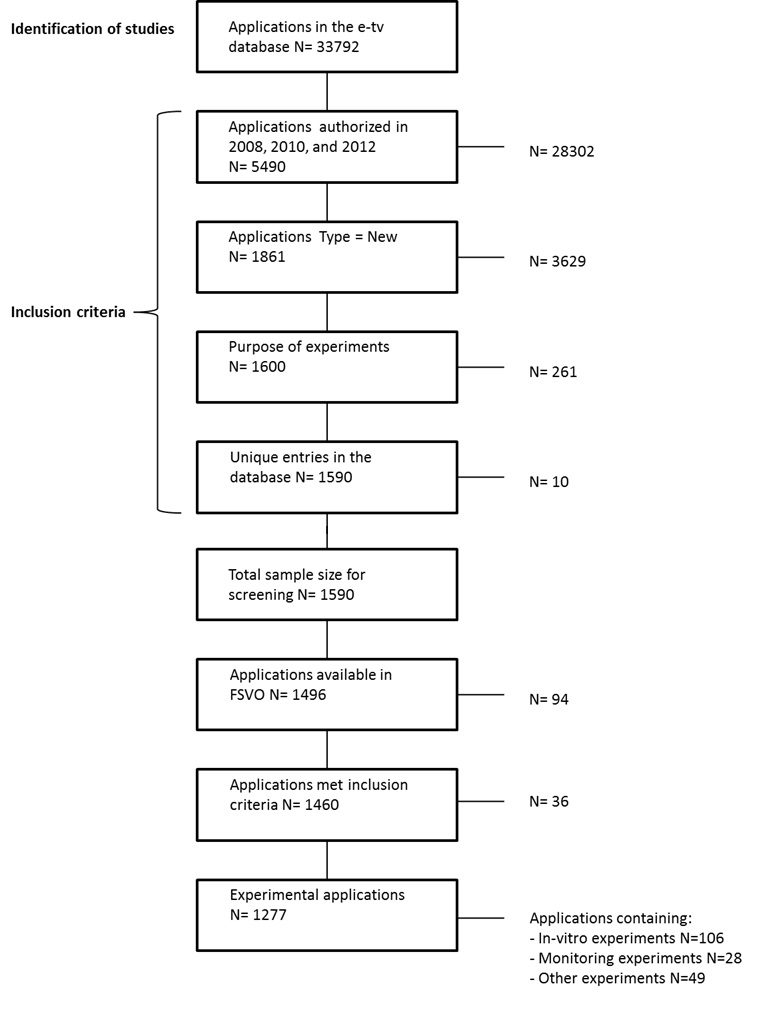

Supplement: S3 Fig — (TIF) [file pbio.2000598.s003.tif]
