# Supplementary material for: Authorization of Animal Experiments Is Based on Confidence Rather than Evidence of Scientific Rigor
Source: PLoS Biol. 2016 Dec 2;14(12):e2000598. doi: 10.1371/journal.pbio.2000598 (PMC5135031; doi:10.1371/journal.pbio.2000598)
Supplement: S1 Text — (PDF) [file pbio.2000598.s004.pdf]

Form-A

No. ....
(to be completed by the authorities)

Application for licence to perform animal experiments

Article 18 Animal Welfare Act (TSchG), article 141 Animal Welfare Ordinance (TSchV), article 30 Animal Experimentation Ordinance (TVV)

---

1 Address of resource manager (institute, company)

E-mail; tel no.

---

2 Address of cantonal authority

---

3 **TITLE OF PROJECT**

---

31 Field of study or area of application:

---

32 ☐ *[N]* new application
☐ *[F]* application for renewal (no.)
☐ *[E]* supplementary application (no.)
In the case of supplementary applications, the requested amendments must be summarized in keywords, the scientific rationale must be indicated in §44.2 and it must be indicated which numbers in the application are affected. The amendments are described alongside the various numbers and must be clearly highlighted versus the existing text:

The modification refers exclusively: ☐ Animal number / species; ☐ Personnel ☐ Validity / extension ☐ Method

---

33

**ANIMAL SPECIES (strain)**	**Total number** per application	**Origin*** (a-c)

*Origin: (a): from previous experiment, please specify licence number:
(b): licensed laboratory animal husbandry in Switzerland (incl. own husbandry): licence number: or laboratory animal breeder or dealer abroad (art. 118 para. 1, TSchV);
(c): other origin, please specify:

Names and addresses of suppliers:

34.1 **Location of animals:** address, room number **licence number of laboratory animal husbandry:**

34.2 **Location of the experiments:** address, room number

34.3 **Intercantonal experiment:** yes ☐ no ☐
if so, which other canton(s):

34.4 **Use of genetically modified animals*** : yes ☐ no ☐
*Data sheets for genetically modified lines and strained mutants must be enclosed (additionally for strained lines: decision number).

---

35 **Maximum prospective degree of severity**: (for details, see para 56.4)

---

36 **Duration of project:**
Date of proposed start:

---

37 **List of persons who perform or lead measures and procedures in the context of the experiment:**

The annex to this application "Persons involved and certificates of education and training" must be duly filled and copies of education and trainings have to be joined.
Persons who lead or perform experiments must satisfy the educational and further training requirements set forth in art. 132 and 134, para. 1 TSchV and Chapter 3 of the ordinance of the FDEA on qualifications in animal husbandry.

38 The undersigned **resource manager** (art. 129, 130, TSchV) confirms that the persons named in the appendix are familiar with the regulations of the TSchG and TSchV applicable to animal experiments and that they satisfy the educational and further training requirements (art. 130 para. d TSchV).

Place and date Name and signature

39 **Study director**: if several people perform this function**,** their areas of responsibility must be defined in the appendix. **Signature of principal study director**: (art. 131, 132 TSchV):

Place and date Name and signature

Deputy study director (requirements as stipulated in art. 129 para. 2 TSchV, art. 30e TVV)

Place and date Name and signature

4 **INFORMATION ON THE PURPOSE OF THE EXPERIMENT** (for statistics art. 147 TSchV);
sections 41 - 43 indicate **one only in each case** and, if applicable, add a further note or additional information in the case of detailed questions.

41 The project is associated with

- ☐ biological (including medical) studies in the field of basic research
- ☐ discovery, development, and quality control (excluding safety testing) of products or devices for human and veterinary medicine
- ☐ diagnosis of disease
- ☐ education and training
- ☐ protection of humans, animals and the environment by toxicological or other safety tests

  ... for substances used or intended to be used mainly
  - ☐ as pharmaceutical products (including medical devices)
  - ☐ in agriculture
  - ☐ in industry
  - ☐ in private households
  - ☐ as cosmetics or toiletries
  - ☐ as food additives or
  - ☐ to determine potential or actual hazards of contaminants in the general environment, or
  - ☐ other uses. Please specify:
- ☐ other studies. Please specify:

42 The project is associated with

- ☐ human diseases
  - ☐ cancer (excluding carcinogenicity studies)
  - ☐ cardiovascular diseases
  - ☐ nervous and mental disorders
  - ☐ other human diseases. Please specify:
- ☐ animal diseases. Please specify:
- ☐ no association with human or animal diseases.

43 The project is associated with procedures required by law (registration and licensing regulations):

- ☐ for Switzerland only
- ☐ for other countries only. Please specify:
- ☐ Both. Please specify:

Mention the relevant guidelines or test methods:

- ☐ The project has no association with procedures required by law.

44.1 General description of the aim of the project, the status of knowledge and presentation of what is not yet sufficiently known (for example summary of National Science Foundation application, **one page maximum**)

44.2 Actual question the experiment is designed to answer

5 **INFORMATION ABOUT THE METHOD** (descriptions and note on sections 51 - 58)

51.1 Overview of the project (experimental design, overview of the method, name of animal model where applicable, the course of the project, flowchart showing sequence of events, biometric planning) (details of the method in para 54)

51.2 Reason for selection of method or model, showing its peculiarities / advantages (art. 137 para. 3 TSchV)

51.3 Reason for selecting animal species and, where applicable, for using animals not bred for experimental purposes

52 Preparation of animals for the experiment (initial examination art. 135 para. 3 TSchV; adaptation art. 119 para. 1 TSchV; type of tagging art. 120 TSchV and art. 5 para. 2 TVV (incl. justification for invasive methods), conditioning, feed or water withdrawal, pre-treatment etc.)

53.1 Anaesthesia and/or other means of controlling pain (preparations, doses, route and frequency of administration, period of time etc.) (art. 135 para. 5 TSchV)

53.2 Reason for selection of anaesthesia and/or analgesia or, if applicable, reason not to use such measures

54.1 Type of procedures/manipulations and parameters to be measured in the animal (indicate sequence of events for individual animal/for animal group): surgical procedures (sequence), substance administration (method and site, amount and frequency), infections, physical treatments (radiation, etc.), follow-up examinations, sampling, reaction tests etc. Details may be provided by means of Standard Operating Procedure (SOP))

54.2 Duration of the series of experiments: total duration of experiment / experiment series (tabulated in an appropriate form where useful): full period of experiment for each individual group or animal, incl. period during which the animal is exposed to substances or other noxae. If animals are to be used repeatedly, indicate the interval between experiments

54.3 Number of animals per experiment/series of experiments: number of groups (including all variables, such as doses, periods of time, controls and details on staggering of experiments over time as stipulated in art. 137 para. 4 lit.c TSchV) and numbers of animals per line, per group, sex of animals

54.4 Reason for the planned number of animal per experiment/series of experiments incl. statistical analysis of data (art. 137 para. 4 TSchV)

55 Evaluation of the method (art. 137 para.3 TSchV)

56.1 Expected effect on the health and well-being of the animals (general condition, activity, water and food intake, pain reactions, duration and course of impairment, further behavioural parameters, growth, expected lethality, etc.)

56.2 Monitoring of well-being of the animals (art. 135 para. 4 TSchV):
- by which person(s)
- frequency
- criteria of evaluation
- documentation (e.g. score sheet as stipulated in art. 144 para. 1 TSchV)
- according to the phase of experiment

56.3 Indications concerning stress-reducing measures and criteria for premature discontinuation of the experiment (criteria for discontinuation; art. 135 para. 1 and 8 TSchV) and criteria for renouncement to reuse animals

56.4 Repartition of animals per severity degree (art. 30 j TVV)

57.1 Indicate the name and/or the number of the husbandry licence
(if there is no licence for keeping laboratory animals indicate: housing and husbandry of animals before, during, between and after individual experiments; space provided, cage type incl. number of animals, structuring of enclosure, run, individual or group housing, feeding and occupation, routine inspections by animal technicians, etc.)

57.2 Reason for any deviations from conditions in which animals are kept as defined in the animal protection ordinance in the above-mentioned licence for keeping laboratory animals (e.g: feed withdrawal, immobilization, single housing for social animal species)

---

58 Fate of the animals: utilisation of the animals at the end of the (individual) experience; repeated use in the same or another experiment).
Method of euthanasia (substance, doses, route of administration, etc.)

---

6 **INFORMATION ON THE REASON FOR THE ANIMAL EXPERIMENT**

61 What other experimental methods are known (e.g. from the literature) which would allow corresponding information to be obtained (mention *in vitro* or *in vivo* methods art. 137 para. 2 and 3 TSchV)

---

62 Information on whether the project has been / is being appraised and, if so, by which institution/organization

---

63 Assessment of the importance of the anticipated information or results in relation to the pain, suffering, injury or anxiety experienced by the animals and injury to the dignity of the animal (art. 3 and 19 para. 4 TSchG,). In this process of weighing up the various interests (art. 26 TVV) the desired benefits as stipulated under sections 44.1 and 44.2 must in particular be assessed and weighed against the stress on the animals as stipulated under sections 56.1 – 56.4
