# Supplementary material for: Authorization of Animal Experiments Is Based on Confidence Rather than Evidence of Scientific Rigor
Source: PLoS Biol. 2016 Dec 2;14(12):e2000598. doi: 10.1371/journal.pbio.2000598 (PMC5135031; doi:10.1371/journal.pbio.2000598)
Supplement: S2 Text — (PDF) [file pbio.2000598.s005.pdf]

Checklist - Evaluation of the Scientific Validity of Animal Experiments in Switzerland

National / Cantonal No.: **Assessor:** **Date:**

Time spent on the evaluation: **Language:** **Species:**

Type of experiment: Experimental ☐ in vitro ☐ **No. of experiments:**

Monitoring ☐ other ☐

Questions about internal validity

NA: not applicable

??: ambiguous/incomprehensible

**1 Allocation concealment:**				
1.1 Induction of disease or animal model	NA	Yes	No	??
**2 Blinding:**				
2.1 Conduct of study	NA	Yes	No	??
2.2 Outcome assessment	NA	Yes	No	??
**3 Randomization:**				
3.1 Treatment allocation	NA	Yes	No	??
3.2 Conduct of study	NA	Yes	No	??
**4 Sample size:**				
4.1 Formally calculated (power analysis) *(54.4)*	NA	Yes	No	??
4.2 Based on some reference	NA	Yes	No	??
**5 Eligibility and drop-outs:**				
5.1 Are inclusion or exlusion criteria of animals specified ?	NA	Yes	No	??
5.2 Are termination criteria specified? *(56.3)*	NA	Yes	No	??
**6 Primary outcome variable:**				
6.1 Is the primary outcome variable specified?	NA	Yes	No	??
**7 Statistical analysis:**				
7.1 Is the method of statistical analysis explicitely specified? *(54.4, 55)*	NA	Yes	No	??
7.2 Is the general statistical method specified? *(54.4, 55)*	NA	Yes	No	??

Questions about the accuracy of Form A

**8 Experimental methods:**				
8.1 Are any references or pilot studies mentioned concerning the methods used?	NA	Yes	No	??
**9 Individual identification:**				
9.1 Is the marking or identification method described? *(52)*	NA	Yes	No	??
**10 Animals:**				
10.1 Does total no. of animals (table 33) match sum of animals of all groups? *(54.3)*	NA	Yes	No	??
10.2 Is the no. of groups (incl. control) correctly stated? *(54.3)*	NA	Yes	No	??
10.3 Is the no. of animals per groups correctly stated? *(54.3)*	NA	Yes	No	??
**11 Animal welfare:**				
11.1 Are score sheets or descriptions of assessed parameters available? *(56.2)*	NA	Yes	No	??
**12 Severity:**				
12.1 Are numbers or percentage of animals given per degree of severity? *(56.4)*	NA	Yes	No	??
**13 End of experiment:**				
13.1 Is the fate of the animals at the end of the experiment stated (e.g. further use, killing method, etc.)? *(58)*	NA	Yes	No	??

Comments:
