# Supplementary material for: Authorization of Animal Experiments Is Based on Confidence Rather than Evidence of Scientific Rigor
Source: PLoS Biol. 2016 Dec 2;14(12):e2000598. doi: 10.1371/journal.pbio.2000598 (PMC5135031; doi:10.1371/journal.pbio.2000598)
Supplement: S3 Text — (PDF) [file pbio.2000598.s006.pdf]

ANNEX TO CHECKLIST: DESCRIPTION OF ITEMS AND MODE OF EVALUATION

This annex complements the checklist for the Evaluation of the Scientific Validity of Animal Experiments in Switzerland. It describes all items comprised by the checklist, and the rules according to which they are evaluated. In general, items may be evaluated in terms of (but not all evaluations apply to all items):

- NA: if the item is not applicable to the application under evaluation,
- YES: if the item as described here is met by the application,
- NO: if the item as described here is not met (explicitly, or no information) by the application,
- ??: if it is unclear whether or not the item as described here is met by the application.
  All items evaluated as ?? will be reassessed by the two evaluators to reach a final decision based on mutual agreement.

Descriptors

Type of experiment

Experimental
Refers to all studies in which some sort of experimental treatment is applied to animals.

Monitoring
Refers to studies without experimental treatments applied to animals. This includes mainly monitoring studies of wild animals with or without manipulation of the animals (e.g. capture – re-capture; transponder fitting under anesthesia). Studies belonging to the type *Monitoring* will be marked as such and not further screened.

In vitro
Refers to studies using animals as a source of organs, tissue or other parts, whereby no experimental treatments are applied to the animals except under terminal anaesthesia or post mortem. In vitro studies will not be evaluated. Studies belonging to the type *In vitro* will be marked as such and not further screened.

Species

List of all relevant species names (preferably scientific name) used in the study.

No of experiments

Refers to the number of distinct experiments included in a study. If a distinct number of experiments cannot be determined, indicate the minimum number or the range of the number of experiments.

Questions about internal validity

1 *Allocation concealment*

1.1 Preparation of the animals, induction of disease or animal model

Evaluation for allocation concealment assesses whether the experimenter preparing the animals (e.g. entry health check, inclusion criteria) or inducing the disease or animal model is blind with respect to the future allocation of the animals to the treatment groups of the experiment.

NA:

- If no preparatory activities are performed or animals are not assigned to treatment groups (e.g. study on strain differences where animals are already "assigned" to treatment groups).

YES:

- If the manipulation (preparation, induction of disease or animal model) on the animals is performed prior to their assignment to treatment groups.

NO:

- If the manipulation (preparation, induction of disease or animal model) on the animal is performed after their assignment to treatment groups.
- If the experimenter who performs the manipulation (preparation, induction of disease or animal model) on the animals is aware of their future allocation to treatment groups.
- If no information is provided as to whether the experimenter who performs the manipulation (preparation, induction of disease or animal model) on the animals is aware of their future allocation to treatment groups.

2 *Blinding*

2.1 Conduct of study

Refers to the application of treatments to, and manipulations of, the animals in the course of the study.

NA:

- If blinding is impossible; i.e. if treatment groups differ visibly (e.g. different strains of mouse).

YES:

- If it is clearly stated that the investigator is blind with respect to the treatment group of the animals when manipulating them throughout the course of the experiment.

NO:

- If the investigator is not blind with respect to the treatment group of the animals when manipulating them throughout the course of the experiment.
- If no information is provided indicating that the investigator is blind with respect to the treatment group of the animals when manipulating them throughout the course of the experiment.

2.2 Outcome assessment

Refers to the collection of data from the animals in the course of the study.

NA:

- If blinding is impossible; i.e. if treatment groups differ visibly (e.g. different strains of mouse).

YES:

- If it is explicitly stated (or unmistakable by the described procedure) that the investigator is blind (unaware) with respect to the treatment group of the animals when collecting data from them.

NO:

- If the investigator is not blind with respect to the treatment group of the animals when collecting data from them.
- If no information is provided indicating that the investigator is blind with respect to the treatment group of the animals when collecting data from them.

3 Randomization

3.1 Treatment allocation

Refers to whether or not the animals are allocated randomly to treatment groups.

NA:

- If there are no treatment groups.

YES:

- If the animals are allocated randomly to treatment groups.
- If a study design of the category of "Randomized Block Designs" (e.g. Latin Square, Cross-over, Double-Crossover) is used.

NO:

- If no information is provided indicating that the animals are allocated randomly to treatment groups.

3.2 Conduct of study

Refers to whether or not the order in which animals are manipulated and data collected is randomized or counterbalanced among treatment groups.

NA:

- If the study design does not allow randomization or counterbalancing.

YES:

- If the order in which animals are manipulated and data collected is randomized or counterbalanced among treatment groups.
- If a study design of the category "Randomized Block Designs" (e.g. Latin Square, Cross-over) is used.
- If the order in which animals are manipulated or data collected is determined by the randomization protocol used for treatment allocation.

NO:

- If the order in which animals are manipulated or data collected is not randomized or counterbalanced among treatment groups.
- If no information is provided indicating that the order in which animals are manipulated or data collected is randomized or counterbalanced among treatment groups.

4 Sample size

4.1 Formally calculated

Refers to a formal calculation of sample size based on power analysis.

NA:

- If the study is a single case study.

YES:

- If the method of sample size calculation (incl. key parameters: outcome variable, power, significance level, effect size) is stated explicitly.

NO:

- If the method of sample size calculation is not stated explicitly or relevant key parameters are missing.

4.2 Based on some reference

Refers to reference to previous studies either by the same or other experimenters (e.g. own data, own experience, pilot studies, publications) or to advice from third parties (e.g. statistical service, biostatisticians).

NA:

- If the study is a single case study.

YES:

- If any reference is provided as to how the sample size was determined ("sample size was chosen so as to reach significant results" is not enough).
- If any reference to a sample size calculation is mentioned but key parameters are missing (e.g. "we have done a power analysis")

NO:

- If no reference is provided as to how the sample size was determined.

5 Eligibility and drop-outs

5.1 Inclusion and exclusion criteria

Refers to criteria determining as to whether or not an animal is a suitable subject for the planned study (e.g. health status, body weight or size, performance). These criteria must be assessed prior to the study onset and need to be taken into account when determining sample size.

YES:

- If inclusion and/or exclusion criteria have been specified (explicitly or implicitly).

NO:

- If no inclusion and/or exclusion criteria have been specified.

5.2 Termination criteria

Refers to criteria for excluding animals in the course of a study.

YES:

- If termination criteria have been specified (explicitly or implicitly).

NO:

- If no termination criteria have been specified.

6 Primary outcome variables

6.1 Specification of primary outcome variable

Refers to the main variable considered in the outcome assessment, i.e. to what is being measured. One primary variable (and possibly several secondary variables) need to be determined prior to the study onset and need to be taken into account when determining sample size.

YES:

- If one primary outcome variable is specified or only one is measured.
- If one of the investigated variables is linked to sample size calculation or statistical analysis.

NO:

- If no primary outcome variable is specified.

7 Statistical analyses

7.1 Specification of statistical model

Refers to whether or not the method by which the data are analyzed has been specified and explained in detail. Whether or not the suggested statistical method is appropriate with respect to the study design will be evaluated for a subset of applications for which sufficient detail is available (with support by Christina Nathues). Whether or not an application qualifies for such further evaluation is noted in the comment section of the checklist.

NA:

- If the study is a single case study.

YES:

- If full detail on statistical model is provided (incl. dependent, independent, and random factors).
- If it is stated that descriptive statistics, but no inferential statistics, will be performed, and a justification is provided for this.

NO:

- If the method of statistical analysis is not specified or not enough detail is provided.

7.2 General statistical method

Refers to whether or not the methods by which the data are analyzed is stated in general terms, meaning that only general concepts of statistics are mentioned but no explicit details provided (i.e. statistical analysis without further explanation).

NA:

- If the study is a single case study.

YES:

- If a statistical method is mentioned (e.g. ANOVA, t-test, parametric statistics, “data will be statistically analyzed” is not enough).

NO:

- If no mention is made of the method of statistical analysis or not enough detail is provided (e.g. “statistical tests will be performed”).

Questions about the accuracy of Form A

8 Experimental methods

8.1 Justification of experimental methods

Refers to the scientific justification of the methods being used.

NA:

- If a new method is being developed or tested.

YES:

- If a reference is provided (e.g. “pers. comm.”, “own data”, “standard method”, citations, etc.) supporting the method for the purpose it is intended to be used.

NO:

- If no reference is provided supporting the use of the method.

9 Individual identification

9.1 Marking and tagging methods

Refers to information on the marking or tagging method as required on the application Form A under point 52.

NA:

- If the experimental unit is a group of animals (e.g. cage, aquarium) rather than the individual animal or if animals are singly housed.
- If the animals are farm animals, pet animals, or zoo animals that are either marked by law (e.g. ear tags, chips), or can be identified by individual characteristics.

YES:

- If the marking method is clearly stated (e.g. ear clipping, staining, fur marks, etc.)

NO:

- If no information on marking or tagging is provided.

10 Animals

10.1 Number of animals

Refers to information on the total number of animals as required on the application Form A under point 54.3, in particular whether the total no. of animals (table 33) matches the sum of animals of all groups.

YES:

- If animal numbers mentioned in section 5 of Form A add up to the number(s) provided in table 33.

NO:

- If animal numbers mentioned in section 5 of Form A do not add up to the number(s) provided in table 33.
- If contradictions occur between the number(s) of animals mentioned at different places on Form A.

10.2 Number of groups

Refers to information on the number of treatment groups as required on the application Form A under point 54.3, in particular whether the number of groups (incl. control group(s)) is correctly stated.

NA:

- If there are no treatment groups (e.g. monitoring studies).

YES:

- If the number of groups being studied is explicitly and consistently stated.

NO:

- If the number of groups is not stated.
- If contradictions occur between the numbers of groups mentioned at different places on Form A.

10.3 Number of animals per group

Refers to information on the number of animals per group as required on the application Form A under

point 54.3, in particular whether the number of animals per group (incl. control group(s)) is correctly stated.

NA:

- If there are no treatment groups (e.g. monitoring studies).

YES:

- If the number of animals per group is consistent with the number of groups and the total number of animals.

NO:

- If the number of animals per group is not stated.
- If the number of animals per group is not consistent with the number of groups or the total number of animals.

11 Animal welfare

11.1 Monitoring of animal welfare

Refers to information regarding the monitoring of animal welfare as required on the application Form A under point 56.2.

NA:

- If the animals are wild animals.

YES:

- If detailed score sheets are provided.
- If the variables and scores and the interval at which they are assessed is described in detail (i.e. "the animals will be checked daily by animal care personnel" is not enough).

NO:

- If no or no clear information is provided on the variables and scores and/or the interval at which they are assessed.

12 Severity

12.1 Degree of severity

Refers to information regarding the number(s) of animals per degree of severity as required on the application Form A under point 56.4.

YES:

- If the number(s) of animals per degree of severity is clearly listed and add up to the number(s) of animals listed in table 33.

NO:

- If no information is provided on the degree(s) of severity of the animals used or if the information is incomplete (i.e. does not include all animals listed in table 33).

13 End of experiment

13.1 Fate of the animals

Refers to information regarding the fate of the animals at the end of the study as required on the application Form A under point 58.

YES:

- If the fate of all animals is clearly stated (e.g. method of euthanasia, rehoming, re-use in following studies).

NO:

- If the fate of the animals is not stated or not for all animals listed in table 33.

Comments

In this section specific comments can be made that may be relevant for the analysis of the evaluations. Moreover, a comment should be made if the application is a potential candidate for a more thorough assessment of the statistical methods.
